# Exploring the Role of mRNA Methylation in Insect Biology and Resistance

**DOI:** 10.3390/insects16050463

**Published:** 2025-04-28

**Authors:** Jiayang Zhang, Luobin Lin, Botian Huang, Huoxi Liu, Huaqin Li, Wenmei Wu

**Affiliations:** 1 Guangdong Provincial Key Laboratory of Advanced Drug Delivery, Guangdong Provincial Engineering Centerof Topical Precise Drug Delivery System, School of Life Sciences and Biopharmaceutics, Guangdong Pharmaceutical University, Guangzhou 510006, China; 2112446047@stu.gdpu.edu.cn (J.Z.); 2100920428@gdpu.edu.cn (L.L.); 2112355024@stu.gdpu.edu.cn (B.H.); 2100930434@stu.gdpu.edu.cn (H.L.); 2 School of Health Sciences, Guangzhou Xinhua University, Guangzhou 510520, China

**Keywords:** epigenetic modifications, *N*^6^-methyladenosine (m^6^A), 5-methylcytosine (m^5^C), *N*^1^-methyladenosine (m^1^A), insects, pest control strategies

## Abstract

RNA methylation, characterized by modifications such as *N*^6^-methyladenosine, 5-methylcytosine, and *N*^1^-methyladenosine plays a crucial role in post-transcriptional gene regulation across diverse biological systems. While research on RNA methylation has predominantly focused on mammals, particularly its roles in epigenetic regulation and cancer biology, recent studies in insects have begun to explore their extensive functions in insect physiology. This review examines the mechanisms by which RNA methylation regulates growth, development, reproduction, environmental adaptation, and immune response in insects, providing insights into the biological characteristics of these organisms without prematurely speculating on pest control strategies. It aims to offer valuable insights into the role of RNA methylation in insect biology and resistance.

## Simple Summary

RNA methylation, a process involving chemical modifications like *N*^6^-methyladenosine, plays a crucial role in regulating gene activity after DNA is transcribed into RNA. While earlier studies focused on its roles in mammals, especially in diseases like cancer, recent research highlights its importance in insects. This review explores how RNA methylation influences key aspects of insect life, including growth, development, reproduction, adaptation, and immune response to environmental challenges. This knowledge provides a foundation for future studies in this emerging field, offering potential for new strategies in pest management and insect control.

## 1. Introduction

**Figure 1:**
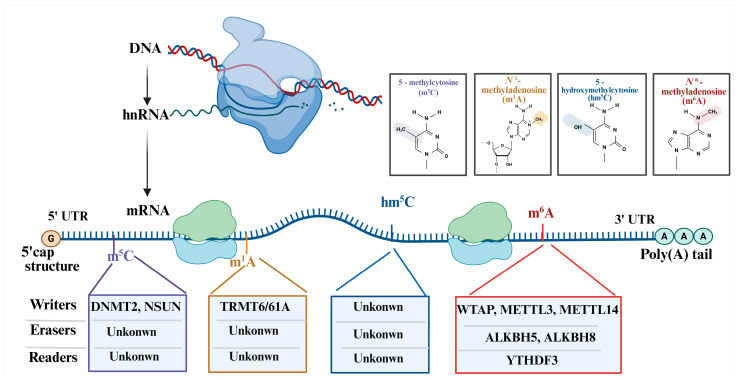
The classification of RNA methylation in insects. Note: The major types of RNA methylation modifications found in insect mRNA are m^5^C, m^1^A, and m^6^A, along with their chemical structures. Methylation events are depicted in distinct transcript regions, including the 5′ untranslated region (UTR), coding sequence, and 3′ UTR. Enzymes involved in the deposition are described as “writers”, “erasers”, and “readers”. Known regulators include DNMT2 and NSUN family members for m^5^C. For m^1^A, TRMT6/61A is responsible for recognition as the “writers”. The m^6^A complex comprises METTL3, METTL14, and WTAP (writers), ALKBH5 (eraser), and YTH domain-containing proteins (readers). Many regulatory components remain unidentified, highlighting the need for further research into insect-specific epitranscriptomic mechanisms.

RNA modifications and their counterparts play a critical role in regulating gene expression by impacting various aspects of RNA metabolisms, including stability, export, translation, and decay [1,2]. This intricate regulatory framework has been traditionally emphasized in mammalian research. However, research on RNA methylation in insects has recently garnered significant attention. RNA methylation plays a pivotal role in the temporal regulation of gene expression, essential for processes such as diapause, metamorphosis, and seasonal adaptation in insects [3,4]. The dynamic interplay within the *N*^6^-methyladenosine (m^6^A) network, along with other modifications such as 5-methylcytosine (m^5^C), *N*^1^-methyladenosine (m^1^A), *N*^6^, 2′-O-dimethyladenosine (m^6^Am), and 5-hydroxymethylcytosine (hm^5^C), implies a complex epitranscriptomic landscape that intricately regulates insect physiology and behavior (Figure 1) [5,6,7,8,9,10]. Considering the substantial influence of pests in agricultural productivity and food security, the investigation of mRNA methylation in pest control strategies is highly crucial [1,11].

The developmental progression of insect cells from the embryonic stage to adulthood is intricately regulated by gene expression. In particular, the m^6^A modification plays a central role, orchestrated by the coordinated actions of the “writer” complex (comprising METTL3, METTL14, and WTAP), “eraser” proteins (notably ALKBH5), and “reader” proteins (such as those from the YTHDF and YTHDC families). This dynamic modulation system exerts a profound influence on the mRNA life cycle, thereby affecting critical cellular processes including proliferation, growth, and cognitive functions [12,13]. This regulatory mechanism extends to insect development, underscoring the necessity for precise gene expression control that is essential to their growth and survival. In the cellular environment, RNA methylation modifications are dynamic, contributing to the precise control of gene expression required for cell fate determination, differentiation, and function [2]. Developmental timing and transitions between life stages are influenced by the presence and recognition of these methylation signals. RNA methylation can activate or repress the translation of key mRNAs. For instance, during periods of rapid cellular division and differentiation, such as larval development or tissue regeneration, RNA methylation patterns undergo significant changes [14]. The cellular stress response is also modulated by RNA methylation. By controlling the stability and translation of stress response mRNAs, methylation marks ensure that insects can rapidly adapt to environmental challenges, including temperature fluctuations, nutrient availability, and immune challenges. Particularly, immune priming in insects refers to the phenomenon where prior exposure to a pathogen enhances the host’s immune response upon subsequent encounters [15]. Epigenetic modifications like m^5^C modifications in rRNA and tRNA have been implicated in regulating protein synthesis during immune responses [10]. Alterations in these modifications can affect the efficiency of immune protein production [5].

As a crucial epitranscriptomic modification, RNA methylation has a complex impact on developmental, neurobiological, and evolutionary dimensions of insect biology. Further investigation into RNA modifications in pests such as *Locusta migratoria*, *Spodoptera frugiperda*, and *Leptinotarsa decemlineata* is crucial, as these insects pose significant agricultural threats due to their extensive crop damage and their increased reliance on chemical pesticides [16,17,18]. By revealing the precise role of RNA methylation in the regulation of pests, it also paves the way for innovative, eco-friendly control methods that mitigate the economic impacts posed by pests like *Helicoverpa armigera* (*H. armigera*) and *Aedes aegypti* (*A. aegypti*) [19,20]. Collectively, these modifications contribute to the nuanced regulatory processes essential for insect adaptation to environmental challenges and stress responses. This review substantiates the integral role of RNA methylation in the regulation of insect growth, immune responses, and reproductive mechanisms.

## 2. m^6^A Methylation in Insects

m^6^A plays an important role in regulating the life cycle and function of mRNAs in insects, mediating effects on splicing, nuclear export, stability, degradation, and translation. In mammals, the ensemble of RNA methyltransferases, including METTL3, METTL14, WTAP, and METTL16, is crucial for RNA metabolism, dictating cellular fate in cancer through vital regulatory processes that determine life or death [21]. This diversity reflects the complexity of mammalian RNA regulation, where methylation affects splicing, stability, translation, and more. In contrast, research in insects has identified a more limited set of methyltransferases, specifically METTL3, METTL14, NSUN2, and WTAP, that catalyze the m^6^A modification [3,22]. This suggests a streamlined yet essential mechanism for regulating the mRNA life cycle and function in insects. For instance, m^6^A modification by METTL3 has been shown to attenuate stress responses in the brain, suggesting a neuroprotective function [23]. The functional interpretation of these modifications is mediated by m^6^A “reader” proteins, such as those from the YTH domain family, which recognize and bind to m^6^A-modified RNAs, influencing their fate and function. The dynamic regulation of m^6^A marks is further nuanced by “eraser” proteins like ALKBH5, which remove methyl groups, allowing for the reversible modulation of RNA messages in response to developmental cues and environmental conditions. A recent study revealed through bioinformatics analysis that ALKBH8, as a key enzyme in insects, is capable of removing methyl marks from RNA, indicating its role as a demethylase [24]. Contrary to its classification as a demethylase in other contexts, in mammals, ALKBH8 catalyzes the methylation of uridine residues at the wobble position (U34) in tRNA molecules [24,25]. This modification is crucial for the proper decoding of mRNA codons during translation. Such a mechanism suggests ALKBH8’s significant influence on insect development, stress response, and adaptation through post-transcriptional regulation. Moreover, ALKBH8, as a potential m^6^A eraser, can reduce the m^6^A levels of *A. aegypti* and *Drosophila melanogaster* (*D. melanogaster*) RNAs, while in mammalian cells, ALKBH8 is known as a tRNA methyltransferase involved in wobble uridine modification and DNA damage survival. Collectively, these activities directly impact RNA’s stability, splicing, and translation by altering m^6^A modification. m^6^A methylation also plays a pivotal role in regulating immune responses in insects. In *Laodelphax striatellus* (*L. striatellus*), m^6^A levels in midgut cells decrease significantly after infection with the Rice Black-Streaked Dwarf Virus (RBSDV) [26]. Silencing of *LsMETTL3* and *LsMETTL14* lowers m^6^A levels and increases viral accumulation, suggesting that m^6^A helps suppress virus replication. In another case, m^6^A targets LsIMPDH, a key enzyme in GTP synthesis. During RSV infection, m^6^A levels on LsIMPDH mRNA increase, while *LsIMPDH* expression and GTP levels decrease, limiting viral replication [27]. These findings reveal that m^6^A methylation not only responds to virus invasion but also helps insects restrict virus spread, offering a potential target for vector-borne virus control.

Recent findings illuminate the pivotal role of m^6^A RNA modification in modulating juvenile hormone (JH) levels, thereby enhancing the fitness of *Plutella xylostella* (*P. xylostella*) in resisting Bacillus thuringiensis (Bt) pathogens through an increased JH titer [28]. Specifically, the m^6^A methyltransferase subunit genes *PxMETTL3* and *PxMETTL14* are identified to repress the expression of JH esterase (JHE), a crucial JH-degrading enzyme, leading to heightened JH levels that mitigate fitness costs associated with robust defense mechanisms against the Bt pathogen [29]. This mechanism, characterized by the downregulation of *PxJHE* through m^6^A modification mediated by PxMETTL3 and PxMETTL14, results in increased m^6^A levels in *PxJHE* mRNA, subsequently reducing *PxJHE* gene expression and elevating JH titer, thus facilitating an optimal balance between growth and Bt pathogen resistance. This insight suggests the critical role of m^6^A in the hormonal regulation of growth–defense trade-offs during host–pathogen interactions, offering potential strategies for managing insect Bt resistance and developing novel pest control approaches. Similarly, a novel study has found that, in *Laodelphax striatellus* (*L. striatellus*) infected with rice stripe virus (RSV), m^6^A modifications on the mRNA of inosine monophosphate dehydrogenase (LsIMPDH), which is essential for GTP synthesis, are elevated, leading to its downregulation and a consequent reduction in GTP levels, which restrict viral replication [27]. The indispensable role of m^6^A in insect biology is underscored by its broad regulatory impact on development, stress response, and viral infection. The evidence increasingly reveals how m^6^A modifications crucially modulate gene expression, cellular metabolism, and host–pathogen interactions. Interestingly, an investigation reveals that m^6^A modulates the cytochrome P450 gene in *Bemisia tabaci* (*B. tabaci*) and *H. armigera*, influencing insecticide resistance [30,31,32]. Previous research has highlighted the significance of specific *P450* genes, notably *CYP6CM1* and *CYP4C64*, in conferring resistance to neonicotinoid insecticides through overexpression [33,34,35]. The regulatory mechanism of m^6^A RNA methylation on these genes remains to be further investigated. Additionally, studies have shown that the knockdown of the methyltransferases *METTL3* and *METTL14* reduces *CYP4C64* expression in resistant *B. tabaci* strains, thereby increasing their susceptibility to thiamethoxam [31]. Additionally, the overexpression of the methyltransferase complex components *WTAP* and *KIAA1429* in these resistant strains suggests their role in enhancing m^6^A methylation and possibly contributes to the regulation of resistance-associated gene expression. It is also revealed that m^6^A plays a crucial role in regulating dopamine synthesis and labor division. The mRNAs encoding dopamine receptor 1 (*Dop1*) and dopamine transporter (*DAT*) undergo m^6^A modification, enhancing their expression and leading to increased dopamine levels. The study further demonstrates that the silencing of the METTLl3 enzyme results in a decrease in the abundance and stability of *Dop1* and *DAT* mRNAs, highlighting the significance of RNA methylation in modulating dopamine synthesis and labor allocation in social insects [36].

In addition to being reported in pests such as *L. striatellus*, *S. frugiperda*, and *B. tabaci*, m^6^A modification also regulates the growth and development processes of model insects like *B. mori* and *D. melanogaster*. In *B. mori* research, knocking down *BmMETTL3* affects various cellular processes including oxidoreductase and transcription regulator activities, as well as cation binding. Subcellular localization experiments in BmN cells reveal the cytoplasmic presence of BmYTHDF3, while BmMETTL3, BmMETTL14, and BmYTHDC are localized in the nucleus [4,13]. This alteration significantly impacts Wnt and Toll/Imd pathways in embryos, emphasizing the essential role of *BmMETTL3* in developmental and physiological regulation and suggesting avenues for genetic interventions in insect biology and biotechnology research. Further investigation reveals that *ie-1* mRNA, among viral genes linked to replication, shows significantly higher m^6^A modification levels. In cells overexpressing *BmYTHDF3*, viral replication decreases in a dose-dependent manner. Conversely, after transfection with si-*BmYTHDF3*, viral replication significantly increases. These findings, demonstrating m^6^A’s role in BmNPV transcripts, provide essential insights into viral mechanics in *B. mori*, expanding the host–virus dynamics and suggesting new approaches for managing viral diseases in sericulture and bioengineering [37]. In *D. melanogaster*, studies have shown that BuGZ, a mitotic effector, exhibits age-related and injury-related condensation in intestinal stem cell nuclei during interphase, with the m^6^A reader YT521-B acting as a key downstream effector [38]. This relationship indicates a complex interaction between BuGZ condensation and m^6^A pathways. Additionally and crucially, the interaction between the YT521-B promoter or m^6^A writer IME4/METTL14 and BuGZ regulates BuGZ’s coacervation, implying that these elements can significantly alter the phase transition of transcription factors [39]. These findings illuminate how epigenetic factors and nuclear architecture collaboratively influence stem cell behavior and tissue repair, providing fresh insights into the processes of aging and regeneration. Another recent study showed that *METTL3* knockdown increased m^6^A target expression and enhanced its stress resistance in *D. melanogaster*, suggesting that *YTHDC1* levels decreased after *METTL3* knockdown [23]. This resilience, also observed with *YTHDC1* knockdown, implies that m^6^A modifications dampen the brain’s stress response. These insights open avenues for therapeutic exploration targeting m^6^A pathways in stress-related conditions. On the other hand, 5′UTR METTL3-dependent m^6^A is enriched in transcripts of neuronal processes and signaling pathways that increase upon stress. *METTL3* knockdown results in increased levels of m^6^A targets and confers resilience to stress in *D. melanogaster*; this result suggests that m^6^A modification dampens the brain’s biological response to stress [23]. Furthermore, the sex determination pathway in *D. melanogaster* involves m^6^A regulation, as evidenced by research on METTL14 and its involvement in the RNA methylation complex. In *Brachionus plicatilis*, knockdown of *METTL3* results in decreased fecundity and premature senescence of rotifers, and RT-qPCR analysis indicates a role for m^6^A in the nonhomologous end-joining pathway of DNA double-strand break repair [24,40]. This complex navigates the transcriptome by attaching m^6^A marks to adenosines within specific RNA consensus sequences (Table 1) [41].

This integration of m^6^A into critical biological pathways underscores the complexity of epigenetic regulation, which highlights the potential for further research in unraveling the multiple roles of RNA modifications in developmental and evolutionary contexts. Understanding m^6^A’s influence on xenobiotic metabolism genes might open pathways for novel insecticide resistance management strategies.

## 3. m^5^C Methylation in Insects

In insects, m^5^C methylation by RNA methyltransferases, particularly NOL1/NOP2/Sun domain (NSUN) family proteins and DNA methyltransferase 2 (DNMT2), is pivotal for RNA stability, translation, and splicing, affecting gene expression and cellular differentiation [55]. In addition, m^5^C plays a critical role in immune memory within generations through immune priming in *Tenebrio molitor* (*T. molitor*) rather than DNA methylation. The reduced RNA methylation in primed insects suggests that RNA methylation may regulate the activation of immune response genes or proteins involved in immune defense [15]. In *D. melanogaster*, embryos deficient in maternal mRNA m^5^C exhibited delays in the cell cycle and were unable to properly initiate the maternal-to-zygotic transition, highlighting the crucial role of maternal mRNA m^5^C modifications mediated by NSUN2 and NSUN6 [5].

DNMT2, traditionally seen as a DNA methyltransferase, plays a crucial role in the epitranscriptomic regulation of insects by methylating tRNAs. DNMT2 enhances stability and function in protein synthesis. While NSUN proteins methylate various RNA species, DNMT2 primarily targets RNA, revealing the intricacy of epigenetic control in insect development, physiology, and adaptation. This highlights the importance of dissecting NSUN and DNMT2 functions in m^5^C methylation for understanding the complex regulation of gene expression and the conservation of epigenetic mechanisms [56]. Specifically, enzymes like NSUN2 methylate certain cytosines in RNA molecules, including tRNA and long non-coding RNAs (lncRNAs), to significantly enhance their stability and functionality, thereby influencing protein synthesis and gene regulation [5]. This methylation alters RNA interactions, folding, and recognition by proteins, underscoring RNA modifications in cellular regulation and homeostasis. More importantly, the dual role of DNMT2, affecting both DNA and RNA, reveals the intricate and crucial nature of methylation processes within biological systems [57]. In the whitefly *B. tabaci*, a study revealed that *DNMT1* knockdown reduces m^5^C levels in the ovary, upregulating the *ftz-f1* gene during choriogenesis [58]. This leads to hypomethylation at the *ftz-f1* promoter, prolonging its expression, which in turn causes elevated 20-hydroxyecdysone levels and overactivates the *Mmp1* gene, therefore protecting tRNAs from degradation. This precise regulation underscores the conservation of methylation mechanisms and their significance in cellular defense and RNA integrity.

The interaction of m^5^C-modified RNAs with reader proteins is pivotal in affecting RNA stability, localization, translation, and splicing. A comprehensive bioinformatics study identified that RNA m^5^C sites across *Homo sapiens*, *Arabidopsis thaliana*, *Mus musculus*, *D. melanogaster*, and *Danio rerio* offer novel perspectives in pest control [59]. The predicted universality of RNA *N*^5^-methylcytosine sites across these species confirms the pervasive role of m^5^C modifications not only in insects but also in a broader range of eukaryotes. This analysis points to the indispensable role of m^5^C in biological systems, emphasizing the evolutionary continuity of epigenetic regulation. For instance, studies revealed that Ypsilon schachtel (YPS), a homolog of human Y box binding protein 1 (YBX1), promotes germ line stem cell (GSC) maintenance, proliferation, and differentiation in the *D. melanogaster* ovary by preferentially binding to m^5^C-containing RNAs [60,61]. Another study suggests that YPS is genetically demonstrated to function intrinsically for GSC maintenance, proliferation, and progeny differentiation in the *D. melanogaster* ovary, and human YBX1 can functionally replace YPS to support normal GSC development [61]. Therefore, overexpression of YPS and YBX1 proteins disrupts GSC development, which means m^5^C RNA modification plays an important role in adult stem cell development. A study on honeybees treated with fipronil, known for its high toxicity to honeybees and aquatic organisms, provided additional evidence for the regulatory role of RNA methylation in response to external stressors [62]. This study found that m^5^C methyltransferases (including AmNOP2, AmNSUN5, AmTET1, and AmYBX1) are likely to regulate fipronil detoxification in honeybees. Further studies should explore the applicability of RNA methylation in assessing pesticide risks to honeybees at both individual and colony levels.

## 4. Other Types of RNA Methylation in Insects

Methylation at the N1 position of adenosine introduces a positive charge on the nitrogen atom, significantly altering the properties of the modified nucleotide and affecting RNA structure and function [8]. The m^1^A modification is catalyzed by a specific class of methyltransferases, which recognize target adenosines within RNA and facilitate the transfer of a methyl group from S-adenosylmethionine to the N1 position [63,64]. However, emerging evidence suggests that m^1^A modifications also occur in mRNA and may have significant implications for mRNA stability, translation efficiency, and the cellular stress response [65]. In tRNAs, m^1^A modification is critical for maintaining the correct tRNA conformation, enabling accurate and efficient amino acid incorporation during protein synthesis [66]. Other studies have shown that tRNA in insect viruses also contains m^1^A modifications, indicating that tRNA fragments are selectively packaged. This is related to virus infection and host adaptation, providing new insights for further research on the role of m^1^A in insect physiological regulation and pest management [67]. Given its impact on RNA structure and translation, m^1^A could influence the temporal and spatial expression of key developmental genes. Moreover, the involvement of m^1^A in the cellular stress response suggests a potential role in developmental plasticity, allowing insects to modify developmental trajectories in response to environmental cues [68]. This adaptive capacity is especially relevant for insects facing habitat changes, fluctuating food resources, or climate variability, highlighting the ecological and evolutionary significance of RNA methylation.

Eukaryotic mRNAs undergo modification at the 5′ end, where a methylated guanosine (m^7^G) is linked to the nucleotide at the transcription start site (TSS). This TSS nucleotide is 2′-O-methylated (Nm) by CMTR1 across various organisms, from insects to humans. In mammals, the adenosine at the TSS can undergo further *N*^6^-methylation by the RNA polymerase II phosphorylated CTD-interacting factor 1, resulting in the formation of m^6^Am [69]. The m^6^Am modification represents another layer of complexity in RNA methylation. m^6^Am is found at the first nucleotide following the 7-methylguanosine cap of mRNA, where it can influence mRNA stability and translation efficiency [70]. The synthesis of m^6^Am is thought to be carried out by a subset of the m^6^A methylation machinery, possibly involving METTL3 in conjunction with others yet to be fully identified. Therefore, the specific biological roles and regulatory mechanisms of m^6^Am in insects are not well characterized. However, the presence of m^6^Am suggests a conserved evolutionary mechanism for fine-tuning gene expression post-transcriptionally, particularly in the regulation of cap-proximal RNA features [71]. The comparative study of m^6^A and m^6^Am methylation between insects and mammals promises to deepen our knowledge of evolutionary biology, with significant implications for pest management, agriculture, and biotechnology.

The presence of hm^5^C as part of a demethylation pathway points to a sophisticated level of epitranscriptomic control, where modifications are not only added but can also be actively removed or converted into different marks, offering a mechanism for rapid adaptation and modulation of gene expression [72]. The conversion of m^5^C to hm^5^C further adds to the regulatory complexity, mediated by enzymes like the ten-eleven translocation family, enhancing RNA function refinement and interaction dynamics [73]. This conversion underscores the sophisticated regulation of RNA processes, warranting further exploration. The presence of hm^5^C indicates a conserved RNA modification pathway, emphasizing the importance of RNA modifications in gene expression and adaptation. Further research into hm^5^C formation and its effects will advance our understanding of the epitranscriptome in insect biology [74,75]. This reversibility suggests a dynamic regulatory process that could fine-tune RNA function in response to cellular cues, environmental changes, or developmental stages. Understanding these processes at the molecular level may reveal novel strategies for pest control and contribute to the development of biotechnological applications based on RNA modification (Table 2).

## 5. Discussion and Concluding Remarks

**Figure 2:**
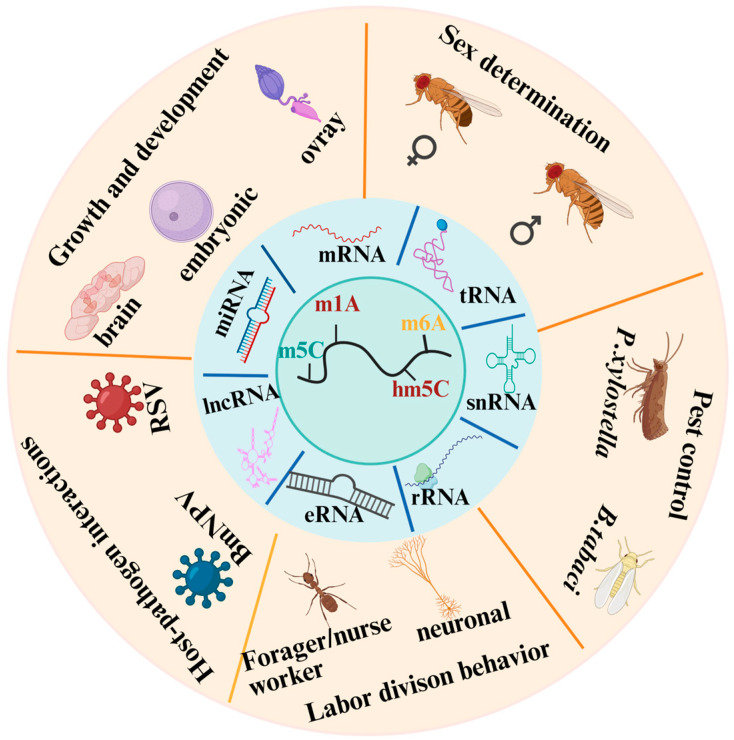
RNA methylation function in insects. Note: RNA methylation, including m^1^A, m^6^A, m^5^C, and hm^5^C, occurs on various RNA types such as mRNA, tRNA, rRNA, lncRNA, miRNA, snRNA, and eRNA in insects. RNA methylation has been shown to regulate embryonic development, neuronal function, ovary maturation, and sex determination. It also plays a key role in caste-specific behaviors such as division of labor between foragers and nurses. RNA methylation is involved in insect–virus interactions, including responses to pathogens like BmNPV and RSV, and holds potential for pest control strategies targeting species such as (*P. xylostella*) and *B. tabaci*. These findings highlight the widespread influence of epitranscriptomic regulation across insect physiology, immunity, and applied entomology.

RNA methylation modification plays a pivotal role in insects’ physiology (Figure 2). Despite the detailed mechanisms of RNA methylation outlined, several questions about the functional implications of this diversity remain unanswered. How does the diversity in RNA methylation patterns affect gene expression and phenotype in insects? Will insecticides developed with this target affect the health of human or beneficial insects? Can such insecticides be widely used in the future? Utilizing CRISPR-Cas9, research demonstrates that enhanced *CYP9A* genes in *Spodoptera exigua* and *Spodoptera frugiperda* enable the metabolism of furanocoumarin, a plant defense compound, along with pyrethroid, avermectin, and oxadiazine insecticides. However, the potential regulation of these phenomena by RNA methylation warrants further investigation [35]. Future studies need to investigate the functional outcomes of these methylation patterns, perhaps through CRISPR-Cas9-mediated editing of methylation sites, to elucidate their roles in insect biology [77]. From a broader perspective of RNA modification, pseudouridine (Ψ) is a prominent post-transcriptional RNA modification found in a variety of RNA species, and by altering RNA structure and stability, it adds another layer of regulation, impacting ribosome function and the fidelity of protein synthesis [78]. This modification enhances the structural integrity of RNA molecules, particularly rRNA and tRNA, which are crucial for efficient protein synthesis and proper ribosomal function [9]. What then is Ψ’s role in RNA modification within the context of those RNA methylations? Research indicates that Ψ modifications in mRNA and lncRNA could influence gene expression patterns crucial for developmental processes. For instance, in *D. melanogaster*, the modulation of Ψ levels in mRNA has been linked to the regulation of circadian rhythms and may affect neuronal function, suggesting a broader role in neurodevelopment and behavior [79]. This modification is catalyzed by pseudouridine synthases (PUS enzymes), which recognize specific uridine residues in RNA and catalyze their conversion to Ψ without the use of a cofactor. Pseudouridylation alters the structure and hydrogen-bonding properties of RNA, influencing its stability, folding, and interaction with proteins, which can significantly impact RNA function across a variety of cellular processes. Investigations into *D. melanogaster* have delineated the neuronal roles of two RNA modification genes, *RluA-1* and *RluA-2*. *RluA-1* is confined to larval sensory neurons, in contrast to the widespread expression of *RluA-2*. Targeted RNAi knockdown of *RluA-1* in nociceptors induces hypersensitivity, a phenotype mirrored by genetic nulls and ameliorated by nociceptor-specific UAS-RluA-1-cDNA expression [80]. Similarly, *RluA-2* loss-of-function mutants exhibit hyperalgesia, highlighting their shared importance in nociceptive modulation. In rRNA, Ψ also plays a critical role in maintaining ribosome integrity and function, facilitating accurate and efficient protein synthesis. Similarly, in tRNA and snRNA, pseudouridylation affects folding and base-pairing. Though the role of Ψ modification in regulating exact cellular movement through specific pathways remains vague in insects, the significance of Ψ in regulating gene expression and maintaining RNA stability is certain. It is worth investigating whether Ψ modification is indispensable or complementary to further regulation in insects.

In insects, RNA methylation typically involves modifications such as m^6^A and m^5^C, which are similar to those in mammals [21]. However, in insects, these modifications may have unique functions during specific developmental stages or in response to environmental stress. For instance, studies in *D. melanogaster* have shown that m^6^A methylation is involved in the sex determination process, a feature not prominently observed in mammals [81,82]. This suggests that m^6^A modification in insects has distinct roles in regulating reproductive development, potentially providing an evolutionary advantage for insects to adapt to diverse environments. In honeybees, m^5^C methylation plays a crucial role in regulating social behavior [14]. m^5^C modification affects the differential development of worker bees and queens. Additionally, some unique RNA modifications in insects are essential for regulating plant–insect interactions, providing new insights into pest management strategies, which are not feasible for study in mammals [83]. Using insect models, researchers can further explore the unique functions of RNA methylation in non-mammalian species and extend these findings to other non-model organisms, thereby enhancing our understanding of the diversity and evolutionary adaptation of RNA modifications across the biological spectrum.

The evolutionary aspect of RNA methylation mechanisms in insects presents another area ripe for investigation. How have these mechanisms evolved across different insect species, and what does this suggest about the evolutionary pressures that have shaped them, especially in social insects [84,85]? Are there conserved methylation patterns among insects that suggest a fundamental biological importance? Comparative genomics and evolutionary biology approaches could shed light on the evolutionary trajectories of RNA methylation mechanisms and their significance in the insect kingdom. Additionally, the link between RNA methylation and insect behavior remains an intriguing avenue for research. How do changes in RNA methylation influence behaviors critical for survival, such as foraging, mating, and social organization? Furthermore, given the crucial role of insects in agriculture, both as pests and pollinators, understanding RNA methylation can have significant biotechnological implications. Can manipulation of RNA methylation patterns be harnessed to develop novel pest control strategies that are more specific and environmentally friendly than current methods [29,85]? Additionally, could enhancing beneficial methylation patterns in pollinators improve their health and efficiency, contributing to agricultural sustainability?

Despite the fundamental role that RNA methylation plays in regulating both pest and economic insects, what specific roles do RNA methylation modifications play in the regulatory mechanism of gene expression associated with insecticide resistance? How does the variability in RNA methylation patterns among different insect species influence their susceptibility or resistance to different insecticides? To what extent can environmental factors influence RNA methylation patterns, and how do these changes contribute to rapid adaptation to pesticides? Are there potential off-target effects of RNA methylation modifications that could impact insect health, behavior, or pesticide resistance? What are the challenges and limitations in current methodologies for studying RNA methylation in insects? Allowing us to answer these questions is spatial transcriptomics, which is an advanced technology allowing simultaneous analysis of gene expression patterns and their spatial distribution in tissues. It provides insights into functional organization and cellular heterogeneity in complex biological systems. Leveraging spatiotemporal omics, its potential applications in RNA methylation research in insects are promising. Spatiotemporal omics can reveal dynamic epigenetic alterations across various physiological states and developmental stages of insects, offering unprecedented insights into their complex regulatory mechanisms. By deciphering these mechanisms, researchers can gain a deeper understanding of insect biology, potentially identifying novel targets for pest control strategies that specifically target essential insect processes analogous to oncogene-induced cellular plasticity in cancer research. When integrated with multimodal data and traditional molecular biology techniques, it would provide comprehensive biological insights, thereby promoting deeper advancements in entomological research [86,87,88].

Addressing the intricate roles of RNA methylation in insect pesticide resistance necessitates innovative methodologies that unravel the molecular complexities of life.

## Figures and Tables

**Table 1:** Mechanistic pathways of m^6^A methylation modulators.

Factor/Enzyme	Species	Pathways	Function	Up/Down
PxMETTL3, PxMETTL14, and YTHDF2 [30,42]	*P. xylostella*	CCR4/NOT complex and HRSP12-RNase P/MRP complex	Regulation of *PxJHE* expression	Down
LsIMPDH [27]	*L. striatellus*	RSV replication	GTP synthesis	Up
SfrMETTL3 and SfrMETTL14 [43]	*S. frugiperda*	/	Embryonic development	/
CYP4C64, WTAP, and KIAA1429 [31]	*B. tabaci*	/	Insecticide resistance	Up
DmMETTL3 [44]	*D. melanogaster*	Wnt	Embryonic development	Down
BmMETTL3 and BmMETTL14 [13,45]	*B. mori*	Wnt and Toll/Imd	Embryonic development	Up
BmYTHDC [13]	*B. mori*	/	Embryonic development	/
BmMETTL3 [46]	*B. mori*	HSC70	Attenuates nucleopolyhedrovirus infection	Up
Juvenile hormone analog and BmMETTL3 [4]	*B. mori*	Biosynthetic pathway of JH III [47]	Embryonic development	Up
N2b2 [48]	*D. melanogaster*	/	Male-specific lethal regulation	Down
Hakai (E3 ubiquitin ligase) [49,50]	*D. melanogaster*	RACK1 Slit-Robo [51]	Defects in morphological traits	Up
ALKBH8	*D. melanogaster* /*Aedes aegypti*	/	mRNA regulation	Down
dTrmt10A [52]	*D. melanogaster*	Neuronal signaling Heat stress pathway	Stress response	Up
Zinc finger CCCH domain-containing protein 13/Flacc [Fl(2)d-associated complex component] [53]	*D. melanogaster*	Wnt/β-catenin signaling p53 pathway [54]	mRNA regulation	Up

**Table 2:** Primal function of epigenetic modification in different insects.

Types of Modification	Different Insects	Function
m^6^A	*B. dorsalis*	Regulates male reproductive system development [76]
m^6^A	*S. invicta*	Regulates dopamine synthesis [36]
m^6^A	*B. mori*	Regulates *Hsc70* expression to suppress BmNPV infection [46]
m^6^A	*L. striatellus*	Regulates GTP levels to inhibit viral replication [27]
m^6^A	*H. armigera*	Regulates expression of *P450* genes, potentially involved in pesticide resistance [30,31,32]
m^6^A	*P. xylostella*	Improving Bt resistance and growth–defense balance [29]
m^6^A	*B. plicatilis*	Reduces fecundity and lifespan; involved in DNA repair [24,40]
m^5^C	*T. molitor*	Enhances immune priming by regulating translation of immune proteins [1]
m^5^C	*A. mellifera*	Associated with RNA metabolism and immune regulation [2]
m^5^C	*B. mori*	Regulates maternal mRNA stability during early embryogenesis [59,60]
m^5^C	*D. melanogaster*	Regulates germ line stem cell development via YPS-m5C RNA interactions [60,61]
m^5^C	*B. tabaci*	Knockdown increases *ftz-f1* expression, which disrupts hormone levels and choriogenesis [62]
m^1^A	*A. mellifera*	Influences selective packaging of tRNA fragments into virions by iflaviruses [67]
m^1^A	*D. melanogaster*	Regulates maternal-to-zygotic transition, including mRNA storage, translational activation, and developmental timing during early embryogenesis [68]

## Data Availability

Data sharing is not applicable to this article as no datasets were generated or analyzed during the current study.

## Abbreviations

m^6^A	*N*^6^-methyladenosine
m^5^C	5-methylcytosine
m^1^A	*N*^1^-methyladenosine
*H. armigera*	*Helicoverpa armigera*
*B. mori*	*Bombyx mori*
*D. melanogaster*	*Drosophila melanogaster*
*T. molitor*	*Tenebrio molitor*
JH	Juvenile hormone
*P. xylostella*	*Plutella xylostella*
*L. striatellus*	*Laodelphax striatellus*
*S. frugiperda*	*Spodoptera frugiperda*
*B. tabaci*	*Bemisia tabaci*
Dop1	Dopamine receptor 1
DAT	Dopamine transporter
NSUN	NOL1/NOP2/Sun domain
DNMT2	DNA methyltransferase 2
lncRNA	Non-coding RNAs
YPS	Ypsilon schachtel
YBX1	Y box binding protein 1
GSC	Germ line stem cell
PUS	Pseudouridine

## References

[1] Sendinc E., Shi Y. (2023). RNA m6A methylation across the transcriptome. Mol. Cell.

[2] Wang M.K., Gao C.C., Yang Y.G. (2023). Emerging Roles of RNA Methylation in Development. Accounts Chem. Res..

[3] Chen Y.H., Jiang T., Yasen A., Fan B.Y., Zhu J., Wang M.X., Shen X.J. (2023). RNA N^6^-methyladenosine of DHAPAT and PAP involves in regulation of diapause of *Bombyx mori* via the lipid metabolism pathway. Bull. Entomol. Res..

[4] Liu S., Tian H., Xu Y., Wang H. (2023). Juvenile hormone regulates silk gene expression by m^6^A RNA methylation. Cell. Mol. Life Sci..

[5] Liu J., Huang T., Chen W., Ding C., Zhao T., Zhao X., Cai B., Zhang Y., Li S., Zhang L. (2022). Developmental mRNA m^5^C landscape and regulatory innovations of massive m^5^C modification of maternal mRNAs in animals. Nat. Commun..

[6] Koyama K., Hayashi G., Ueda H., Ota S., Nagae G., Aburatani H., Okamoto A. (2021). Base-resolution analysis of 5-hydroxymethylcytidine by selective oxidation and reverse transcription arrest. Org. Biomol. Chem..

[7] Anderson B.R., Muramatsu H., Nallagatla S.R., Bevilacqua P.C., Sansing L.H., Weissman D., Kariko K. (2010). Incorporation of pseudouridine into mRNA enhances translation by diminishing PKR activation. Nucleic Acids Res..

[8] Oerum S., Degut C., Barraud P., Tisne C. (2017). m1A Post-Transcriptional Modification in tRNAs. Biomolecules.

[9] Song W., Podicheti R., Rusch D.B., Tracey W.D. (2023). Transcriptome-wide analysis of pseudouridylation in *Drosophila melanogaster*. G3-Genes Genom. Genet..

[10] Bataglia L., Simoes Z., Nunes F. (2021). Active genic machinery for epigenetic RNA modifications in bees. Insect. Mol. Biol..

[11] Jiao Y., Palli S.R. (2023). N^6^-adenosine (m^6^A) mRNA methylation is required for *Tribolium castaneum* development and reproduction. Insect Biochem. Mol..

[12] Zaccara S., Ries R.J., Jaffrey S.R. (2019). Reading, writing and erasing mRNA methylation. Nat. Rev. Mol. Cell Biol..

[13] Liu S.Q., Jia S.Z., Tian H., Li Y.H., Hu K.W., Tao J.G., Lu Y.C., Xu Y.S., Wang H.B. (2023). Evolution of m6A-related genes in insects and the function of METTL3 in silkworm embryonic development. Insect Mol. Biol..

[14] Wang M., Xiao Y., Li Y., Wang X., Qi S., Wang Y., Zhao L., Wang K., Peng W., Luo G.Z. (2021). RNA m^6^A Modification Functions in Larval Development and Caste Differentiation in Honeybee (*Apis mellifera*). Cell Rep..

[15] Castro-Vargas C., Linares-Lopez C., Lopez-Torres A., Wrobel K., Torres-Guzman J.C., Hernandez G.A., Wrobel K., Lanz-Mendoza H., Contreras-Garduno J. (2017). Methylation on RNA: A Potential Mechanism Related to Immune Priming within But Not across Generations. Front. Microbiol..

[16] Piou C., Marescot L. (2023). Spatiotemporal risk forecasting to improve locust management. Curr. Opin. Insect Sci..

[17] Li Y., Wang Z., Romeis J. (2021). Managing the Invasive Fall Armyworm through Biotech Crops: A Chinese Perspective. Trends Biotechnol..

[18] Timani K., Bastarache P., Morin P.J. (2023). Leveraging RNA Interference to Impact Insecticide Resistance in the Colorado Potato Beetle, *Leptinotarsa decemlineata*. Insects.

[19] Quan Y., Wu K. (2023). Managing Practical Resistance of Lepidopteran Pests to Bt Cotton in China. Insects.

[20] Saavedra-Rodriguez K., Campbell C.L., Lozano S., Penilla-Navarro P., Lopez-Solis A., Solis-Santoyo F., Rodriguez A.D., Perera R., Black I.W. (2021). Permethrin resistance in Aedes aegypti: Genomic variants that confer knockdown resistance, recovery, and death. PLoS Genet..

[21] Lin L., Zhao Y., Zheng Q., Zhang J., Li H., Wu W. (2023). Epigenetic targeting of autophagy for cancer: DNA and RNA methylation. Front. Oncol..

[22] Scholler E., Weichmann F., Treiber T., Ringle S., Treiber N., Flatley A., Feederle R., Bruckmann A., Meister G. (2018). Interactions, localization, and phosphorylation of the m^6^A generating METTL3-METTL14-WTAP complex. RNA.

[23] Perlegos A.E., Shields E.J., Shen H., Liu K.F., Bonini N.M. (2022). Mettl3-dependent m^6^A modification attenuates the brain stress response in *Drosophila*. Nat. Commun..

[24] Dai Z., Asgari S. (2023). ALKBH8 as a potential N^6^-methyladenosine (m^6^A) eraser in insects. Insect Mol. Biol..

[25] Honda K., Hase H., Tanikawa S., Okawa K., Chen L., Yamaguchi T., Nakai M., Kitae K., Ago Y., Nakagawa S. (2024). ALKBH8 contributes to neurological function through oxidative stress regulation. Proc. Natl. Acad. Sci. Nexus.

[26] Tian S., Wu N., Zhang L., Wang X. (2021). RNA N^6^ -methyladenosine modification suppresses replication of rice black streaked dwarf virus and is associated with virus persistence in its insect vector. Mol. Plant Pathol..

[27] Zhu M., Wu N., Zhong J., Chen C., Liu W., Ren Y., Wang X., Jin H. (2024). N^6^-methyladenosine modification of the mRNA for a key gene in purine nucleotide metabolism regulates virus proliferation in an insect vector. Cell Rep..

[28] Yang J., Chen S., Xu X., Lin S., Wu J., Lin G., Bai J., Song Q., You M., Xie M. (2023). Novel miR-108 and miR-234 target juvenile hormone esterase to regulate the response of *Plutella xylostella* to Cry1Ac protoxin. Ecotoxicol. Environ. Safe.

[29] Guo Z., Bai Y., Zhang X., Guo L., Zhu L., Sun D., Sun K., Xu X., Yang X., Xie W. (2024). RNA m^6^A Methylation Suppresses Insect Juvenile Hormone Degradation to Minimize Fitness Costs in Response to A Pathogenic Attack. Adv. Sci..

[30] Wang R., Che W., Wang J., Qu C., Luo C. (2020). Cross-resistance and biochemical mechanism of resistance to cyantraniliprole in a near-isogenic line of whitefly *Bemisia tabaci* Mediterranean (Q biotype). Pestic. Biochem. Physiol..

[31] Yang X., Wei X., Yang J., Du T., Yin C., Fu B., Huang M., Liang J., Gong P., Liu S. (2021). Epitranscriptomic regulation of insecticide resistance. Sci. Adv..

[32] Shi Y., Jiang Q., Yang Y., Feyereisen R., Wu Y. (2021). Pyrethroid metabolism by eleven *Helicoverpa armigera* P450s from the CYP6B and CYP9A subfamilies. Insect Biochem. Mol..

[33] Nauen R., Vontas J., Kaussmann M., Wolfel K. (2013). Pymetrozine is hydroxylated by CYP6CM1, a cytochrome P450 conferring neonicotinoid resistance in *Bemisia tabaci*. Pestic. Manag. Sci..

[34] Wang R., Che W., Wang J., Luo C. (2020). Monitoring insecticide resistance and diagnostics of resistance mechanisms in *Bemisia tabaci* Mediterranean (Q biotype) in China. Pesti. Biochem. Physiol..

[35] Shi Y., Liu Q., Lu W., Yuan J., Yang Y., Oakeshott J., Wu Y. (2023). Divergent amplifications of CYP9A cytochrome P450 genes provide two noctuid pests with differential protection against xenobiotics. Proc. Natl. Acad. Sci. USA.

[36] Chen J., Guan Z., Sun L., Fan X., Wang D., Yu X., Lyu L., Qi G. (2024). N^6^-methyladenosine modification of RNA controls dopamine synthesis to influence labour division in ants. Mol. Ecol..

[37] Zhang X., Zhang Y., Pan J., Gong C., Hu X. (2022). Identification and Characterization of BmNPV m6A Sites and Their Possible Roles During Viral Infection. Front. Immunol..

[38] Zhang Q., Deng K., Liu M., Yang S., Xu W., Feng T., Jie M., Liu Z., Sheng X., Chen H. (2023). Phase separation of BuGZ regulates gut regeneration and aging through interaction with m^6^A regulators. Nat. Commun..

[39] Zhou B., Liu C., Xu L., Yuan Y., Zhao J., Zhao W., Chen Y., Qiu J., Meng M., Zheng Y. (2021). N^6^-Methyladenosine Reader Protein YT521-B Homology Domain-Containing 2 Suppresses Liver Steatosis by Regulation of mRNA Stability of Lipogenic Genes. Hepatology.

[40] Zhang Y., Zhou Y., Kan D., Yang Y., Shen J., Han C., Liu X., Yang J. (2023). m6A-mediated nonhomologous end joining (NHEJ) pathway regulates senescence in *Brachionus plicatilis* (*Rotifera*). Arch. Gerontol. Geriatr..

[41] Haussmann I.U., Bodi Z., Sanchez-Moran E., Mongan N.P., Archer N., Fray R.G., Soller M. (2016). m^6^A potentiates Sxl alternative pre-mRNA splicing for robust *Drosophila* sex determination. Nature.

[42] Du H., Zhao Y., He J., Zhang Y., Xi H., Liu M., Ma J., Wu L. (2016). YTHDF2 destabilizes m^6^A-containing RNA through direct recruitment of the CCR4-NOT deadenylase complex. Nat. Commun..

[43] Chen Y., Lai Y., Liu R., Yao L., Yu X.Q., Wang X. (2023). Transcriptome-wide analysis of mRNA N^6^ -methyladenosine modification in the embryonic development of *Spodoptera frugiperda*. Insect Sci..

[44] Sami J.D., Spitale R.C., Cleary M.D. (2022). mRNAs encoding neurodevelopmental regulators have equal N^6^-methyladenosine stoichiometry in *Drosophila* neuroblasts and neurons. Neural Dev..

[45] Li B., Hu P., Huang Z.H., Yang J.Y., Wang J., Xie X.Z., Wang Y.H., Li C.C., Xu J.P. (2023). RNA methyltransferase BmMettl3 and BmMettl14 in silkworm (*Bombyx mori*) and the regulation of silkworm embryonic development. Arch. Insect Biochem..

[46] Chen F., Zhou M., Chen W., Geng W., Lu L., Shen G., Lin P., Xia Q., Zhao P., Li Z. (2025). N^6^-methyladenosine modification of host Hsc70 attenuates nucleopolyhedrovirus infection in the lepidopteran model insect *Bombyx mori*. Int. J. Biol. Macromol..

[47] Van Ekert E., Heylen K., Rouge P., Powell C.A., Shatters R.J., Smagghe G., Borovsky D. (2014). Aedes aegypti juvenile hormone acid methyl transferase, the ultimate enzyme in the biosynthetic pathway of juvenile hormone III, exhibits substrate control. J. Insect Physiol..

[48] Jalloh B., Lancaster C.L., Rounds J.C., Brown B.E., Leung S.W., Banerjee A., Morton D.J., Bienkowski R.S., Fasken M.B., Kremsky I.J. (2023). The *Drosophila* Nab2 RNA binding protein inhibits m^6^A methylation and male-specific splicing of Sex lethal transcript in female neuronal tissue. eLife.

[49] Wang Y., Zhang L., Ren H., Ma L., Guo J., Mao D., Lu Z., Lu L., Yan D. (2021). Role of Hakai in m^6^A modification pathway in *Drosophila*. Nat. Commun..

[50] Bawankar P., Lence T., Paolantoni C., Haussmann I.U., Kazlauskiene M., Jacob D., Heidelberger J.B., Richter F.M., Nallasivan M.P., Morin V. (2021). Hakai is required for stabilization of core components of the m^6^A mRNA methylation machinery. Nat. Commun..

[51] Liu M., Jiang K., Lin G., Liu P., Yan Y., Ye T., Yao G., Barr M.P., Liang D., Wang Y. (2018). Ajuba inhibits hepatocellular carcinoma cell growth via targeting of beta-catenin and YAP signaling and is regulated by E3 ligase Hakai through neddylation. J. Exp. Clin. Cancer Res..

[52] Perlegos A.E., Quan X., Donnelly K.M., Shen H., Shields E.J., Elashal H., Fange L.K., Bonini N.M. (2023). dTrmt10A impacts Hsp70 chaperone m^6^A levels and the stress response in the *Drosophila* brain. Sci. Rep..

[53] Knuckles P., Lence T., Haussmann I.U., Jacob D., Kreim N., Carl S.H., Masiello I., Hares T., Villasenor R., Hess D. (2018). Zc3h13/Flacc is required for adenosine methylation by bridging the mRNA-binding factor Rbm15/Spenito to the m^6^A machinery component Wtap/Fl(2)d. Genes Dev..

[54] Lin X., Wang F., Chen J., Liu J., Lin Y.B., Li L., Chen C.B., Xu Q. (2022). N^6^-methyladenosine modification of CENPK mRNA by ZC3H13 promotes cervical cancer stemness and chemoresistance. Mil. Med. Res..

[55] Chen Y.S., Yang W.L., Zhao Y.L., Yang Y.G. (2021). Dynamic transcriptomic m^5^C and its regulatory role in RNA processing. Wiley Interdiscip. Rev. RNA.

[56] Bohnsack K.E., Hobartner C., Bohnsack M.T. (2019). Eukaryotic 5-methylcytosine (m^5^C) RNA Methyltransferases: Mechanisms, Cellular Functions, and Links to Disease. Genes.

[57] Cunningham C.B., Shelby E.A., McKinney E.C., Simmons A.M., Moore A.J., Moore P.J. (2024). An association between Dnmt1 and Wnt in the production of oocytes in the whitefly *Bemisia tabaci*. Insect Mol. Biol..

[58] Abbas Z., Rehman M.U., Tayara H., Zou Q., Chong K.T. (2023). XGBoost framework with feature selection for the prediction of RNA N5-methylcytosine sites. Mol. Ther..

[59] Mansfield J.H., Wilhelm J.E., Hazelrigg T. (2002). Ypsilon Schachtel, a *Drosophila* Y-box protein, acts antagonistically to Orb in the oskar mRNA localization and translation pathway. Development.

[60] Thieringer H.A., Singh K., Trivedi H., Inouye M. (1997). Identification and developmental characterization of a novel Y-box protein from *Drosophila melanogaster*. Nucleic Acids Res..

[61] Zou F., Tu R., Duan B., Yang Z., Ping Z., Song X., Chen S., Price A., Li H., Scott A. (2020). *Drosophila* YBX1 homolog YPS promotes ovarian germ line stem cell development by preferentially recognizing 5-methylcytosine RNAs. Proc. Natl. Acad. Sci. USA.

[62] Fan M., Qi S., Jiang N., Li Q., Zhao L., Wu L., Huang S., Wang M. (2023). Exploring RNA methylation as a promising biomarker for assessing sublethal effects of fipronil on honeybees (*Apis mellifera* L.). Ecotoxicol. Environ. Safe.

[63] Degut C., Roovers M., Barraud P., Brachet F., Feller A., Larue V., Al R.A., Caillet J., Droogmans L., Tisne C. (2019). Structural characterization of *B. subtilis* m^1^A_22_ tRNA methyltransferase TrmK: Insights into tRNA recognition. Nucleic Acids Res..

[64] Adami R., Bottai D. (2020). S-adenosylmethionine tRNA modification: Unexpected/unsuspected implications of former/new players. Int. J. Biol. Sci..

[65] Safra M., Sas-Chen A., Nir R., Winkler R., Nachshon A., Bar-Yaacov D., Erlacher M., Rossmanith W., Stern-Ginossar N., Schwartz S. (2017). The m^1^A landscape on cytosolic and mitochondrial mRNA at single-base resolution. Nature.

[66] Liu F., Clark W., Luo G., Wang X., Fu Y., Wei J., Wang X., Hao Z., Dai Q., Zheng G. (2016). ALKBH1-Mediated tRNA Demethylation Regulates Translation. Cell.

[67] Simonova A., Romanska V., Benoni B., Skubnik K., Smerdova L., Prochazkova M., Spustova K., Moravcik O., Gahurova L., Paces J. (2022). Honeybee Iflaviruses Pack Specific tRNA Fragments from Host Cells in Their Virions. Chembiochem.

[68] Aviles-Pagan E.E., Orr-Weaver T.L. (2018). Activating embryonic development in *Drosophila*. Semin. Cell Dev. Biol..

[69] Franco G., Taillebourg E., Delfino E., Homolka D., Gueguen N., Brasset E., Pandey R.R., Pillai R.S., Fauvarque M.O. (2023). The catalytic-dead Pcif1 regulates gene expression and fertility in *Drosophila*. RNA.

[70] Angelova M.T., Dimitrova D.G., Da S.B., Marchand V., Jacquier C., Achour C., Brazane M., Goyenvalle C., Bourguignon-Igel V., Shehzada S. (2020). tRNA 2′-O-methylation by a duo of TRM7/FTSJ1 proteins modulates small RNA silencing in *Drosophila*. Nucleic Acids Res..

[71] Dohnalkova M., Krasnykov K., Mendel M., Li L., Panasenko O., Fleury-Olela F., Vagbo C.B., Homolka D., Pillai R.S. (2023). Essential roles of RNA cap-proximal ribose methylation in mammalian embryonic development and fertility. Cell Rep..

[72] Shi H., Chai P., Jia R., Fan X. (2020). Novel insight into the regulatory roles of diverse RNA modifications: Re-defining the bridge between transcription and translation. Mol. Cancer.

[73] Zhang X., Zhang Y., Wang C., Wang X. (2023). TET (Ten-eleven translocation) family proteins: Structure, biological functions and applications. Signal Transduct. Target. Ther..

[74] Delatte B., Wang F., Ngoc L.V., Collignon E., Bonvin E., Deplus R., Calonne E., Hassabi B., Putmans P., Awe S. (2016). RNA biochemistry. Transcriptome-wide distribution and function of RNA hydroxymethylcytosine. Science.

[75] Wang S., Xie H., Mao F., Wang H., Wang S., Chen Z., Zhang Y., Xu Z., Xing J., Cui Z. (2022). N^4^-acetyldeoxycytosine DNA modification marks euchromatin regions in *Arabidopsis thaliana*. Genome Biol..

[76] Zhang Q., Li Z., Qiao J., Zheng C., Zheng W., Zhang H. (2025). METTL3/METTL14-mediated RNA m^6^A modification is involved in male reproductive development in *Bactrocera dorsalis*. Insect Science.

[77] Sieriebriennikov B., Reinberg D., Desplan C. (2021). A molecular toolkit for superorganisms. Trends Genet..

[78] Tortoriello G., de Celis J.F., Furia M. (2010). Linking pseudouridine synthases to growth, development and cell competition. FEBS J..

[79] Vicidomini R., Di Giovanni A., Petrizzo A., Iannucci L.F., Benvenuto G., Nagel A.C., Preiss A., Furia M. (2015). Loss of *Drosophila* pseudouridine synthase triggers apoptosis-induced proliferation and promotes cell-nonautonomous EMT. Cell Death Dis..

[80] Song W., Ressl S., Tracey W.D. (2020). Loss of Pseudouridine Synthases in the RluA Family Causes Hypersensitive Nociception in *Drosophila*. G3-Genes Genom. Genet..

[81] Lence T., Akhtar J., Bayer M., Schmid K., Spindler L., Ho C.H., Kreim N., Andrade-Navarro M.A., Poeck B., Helm M. (2016). m^6^A modulates neuronal functions and sex determination in *Drosophila*. Nature.

[82] Guo J., Tang H.W., Li J., Perrimon N., Yan D. (2018). Xio is a component of the *Drosophila* sex determination pathway and RNA N^6^-methyladenosine methyltransferase complex. Proc. Natl. Acad. Sci. USA.

[83] Li S., Tan X.Y., He Z., Shen C., Li Y.L., Qin L., Zhao C.Q., Luo G.H., Fang J.C., Ji R. (2025). The dynamics of N^6^-methyladenine RNA modification in resistant and susceptible rice varieties responding to rice stem borer damage. Insect Sci..

[84] Honeybee Genome Sequencing Consortium (2006). Insights into social insects from the genome of the honeybee Apis mellifera. Nature.

[85] Muthu L.B.C., Murugan M., Pavithran S., Naveena K. (2023). Enthralling genetic regulatory mechanisms meddling insecticide resistance development in insects: Role of transcriptional and post-transcriptional events. Front. Mol. Biosci..

[86] Wang Y., Liu B., Zhao G., Lee Y., Buzdin A., Mu X., Zhao J., Chen H., Li X. (2023). Spatial transcriptomics: Technologies, applications and experimental considerations. Genomics.

[87] Liu L., Chen A., Li Y., Mulder J., Heyn H., Xu X. (2024). Spatiotemporal omics for biology and medicine. Cell.

[88] Li B., Zheng L., Yang J., Qu L. (2024). Targeting oncogene-induced cellular plasticity for tumor therapy. Adv. Biotechnol..

